# Exchanging Advertisements

**Published:** 1877-02-20

**Authors:** 


Exchanging Advertisements.—Several of our cotemporaries have proposed an exchange of advertisements, We would gladly accept the proposition from our editorial brethren of other journals, but our arrangements at present will not admit of it. After a time, we hope to be able to extend as well as avail ourselves of these courtesies.

